# The Effects of H_2_S on the Activities of CYP2B6, CYP2D6, CYP3A4, CYP2C19 and CYP2C9 *in Vivo* in Rat

**DOI:** 10.3390/ijms141224055

**Published:** 2013-12-10

**Authors:** Xianqin Wang, Anyue Han, Congcong Wen, Mengchun Chen, Xinxin Chen, Xuezhi Yang, Jianshe Ma, Guanyang Lin

**Affiliations:** 1 Analytical and Testing Center, Wenzhou Medical University, Wenzhou 325035, China; E-Mails: lankywang@foxmail.com (X.W.); vincehan@163.com (A.H.); bluce494949@163.com (C.W.); cmcwzmc@163.com (M.C.); sunshine.724@163.com (X.C.); jianshema@gmail.com (J.M.); 2 Department of pharmacy, the First Affiliated Hospital, Wenzhou Medical University, Wenzhou 325000, China; E-Mail: yangxuezhi1977@163.com

**Keywords:** CYP450, H_2_S, Cocktail, LC-MS

## Abstract

Hydrogen sulfide (H_2_S) is a colorless, flammable, extremely hazardous gas with a “rotten egg” smell. The human body produces small amounts of H_2_S and uses it as a signaling molecule. The cocktail method was used to evaluate the influence of H_2_S on the activities of CYP450 in rats, which were reflected by the changes of pharmacokinetic parameters of five specific probe drugs: bupropion, metroprolol, midazolam, omeprazole and tolbutamide, respectively. The rats were randomly divided into two groups, control group and H_2_S group. The H_2_S group rats were given 5 mg/kg NaHS by oral administration once a day for seven days. The mixture of five probes was given to rats through oral administration and the blood samples were obtained at a series of time-points through the caudal vein. The concentrations of probe drugs in rat plasma were measured by LC-MS. In comparing the H_2_S group with the control group, there was a statistically pharmacokinetics difference for midazolam and tolbutamide; the area under the plasma concentration-time curve (*AUC*) was decreased for midazolam (*p* < 0.05) and increased for tolbutamide (*p* < 0.05); while there was no statistical pharmacokinetics difference for bupropion, metroprolol and omeprazole. H_2_S could not influence the activities of CYP2B6, CYP2D6 and CYP2C19 in rats, while H_2_S could induce the activity of CYP3A4 and inhibit the activity of CYP2C9 in rats.

## 1. Introduction

H_2_S is a natural decaying product of organic matter, which is highly toxic as a result of environmental and industrial exposure [1]. It is one of the major toxic gases in forensic practices and is absorbed by the upper respiratory tract mucosa. The results from an increasing number of studies are reporting that H_2_S is produced endogenously in various parts of the body and that H_2_S is involved in many physiological functions in mammals, especially in the nervous system and cardiovascular system [2–4]. H_2_S has been referred to as the third gaseous signaling molecule alongside nitric oxide (NO) and carbon monoxide (CO) [2,5,6]. It is involved in the relaxation of smooth muscle and as a vasodilator [7] and is also active in the brain, where it increases the response of the NMDA receptor and facilitates long term potentiation [8]. H_2_S is recognized as potentially protecting against cardiovascular disease [7,9].

It may cause a change in the activity of metabolic enzymes *in vivo* after administering H_2_S. CYP450 are the main metabolic enzymes at the *in vivo* level; therefore, to investigate the effects of H_2_S on the CYP450 activity is very important for further chemotherapy. Cytochrome P450 enzymes comprise a superfamily of hemoproteins, and three families (CYP1, CYP2, and CYP3) are mainly involved in the metabolism of drugs in both humans and rats [10]. They are a large and diverse group of enzymes that metabolize thousands of endogenous and exogenous chemicals. The most common reaction catalyzed by CYP, is a monooxygenase reaction; it plays an important role in Phase I metabolism. Among the CYP isoforms, families 1 through 3 are the major enzymes involved in drug metabolism, accounting for about 75% of the total number of different metabolic reactions [11]. More than 90%-marketed drugs are metabolized by the CYP1A2, 2D6, 2C9, 2C19 and 3A isoforms [12].

In order to assess various individual CYP450 activities, probe drugs have been widely used in many clinical investigations in the field of drug metabolism and pharmacogenetics [13–15]. Probe drug is one kind of compound specially catalyzed by CYP isoforms, and the activities of CYP isoforms can be reflected by the metabolic rate of probe drug. As several CYP isoforms involved in drug metabolism, the cocktail approach was developed.

In this paper, the cocktail probe drugs approach is used to evaluate the induction or inhibition effects of H2S on the activities of rats CYP450 isoforms such as CYP2B6, CYP2D6, CYP3A4, CYP2C19 and CYP2C9 in rats, which are reflected by the changes of pharmacokinetic parameters of five specific probe drugs (bupropion, metroprolol, midazolam, omeprazole and tolbutamide), then provide guidance for rational clinical oral administration after administration of NaHS.

## 2. Results and Discussion

### 2.1. Method Validation

Calibration curves for five probe drugs were generated by linear regression of peak area ratios against concentrations, respectively. The calibration plot of the probe drugs in the range of 10–2000 ng/mL were, *y* = 0.0017*x* + 0.0381 (*r* = 0.9982) for bupropion, *y* = 0.0035*x* + 0.092 (0.9962) for metroprolol, *y* = 0.0037*x* + 0.1538 (*r* = 0.9979) for midazolam, *y* = 0.0006*x* + 0.0321 (*r* = 0.9961) for omeprazole, *y* = 0.0008*x* − 0.0037 (*r* = 0.9991) for tolbutamide. Each probe drug peak area ratio with concentration has a good linear relationship. The lower limit of quantification (LLOQ) for each probe drug in rat plasma was 10 ng/mL.

The relative standard deviation (RSD%) of the five probe drugs in low, medium and high three concentrations were less than 15%. The intra-day and inter-day relative error (RE%) ranged from −15% to 13%. The results demonstrate that the values were within the acceptable range and the method was accurate and precise. The extraction recoveries were ranged from 80% to 95%. The results of matrix effect, the percent nominal concentration were more than 85% or less than 110%.

### 2.2. Pharmacokinetic Study

**Figure 1.**
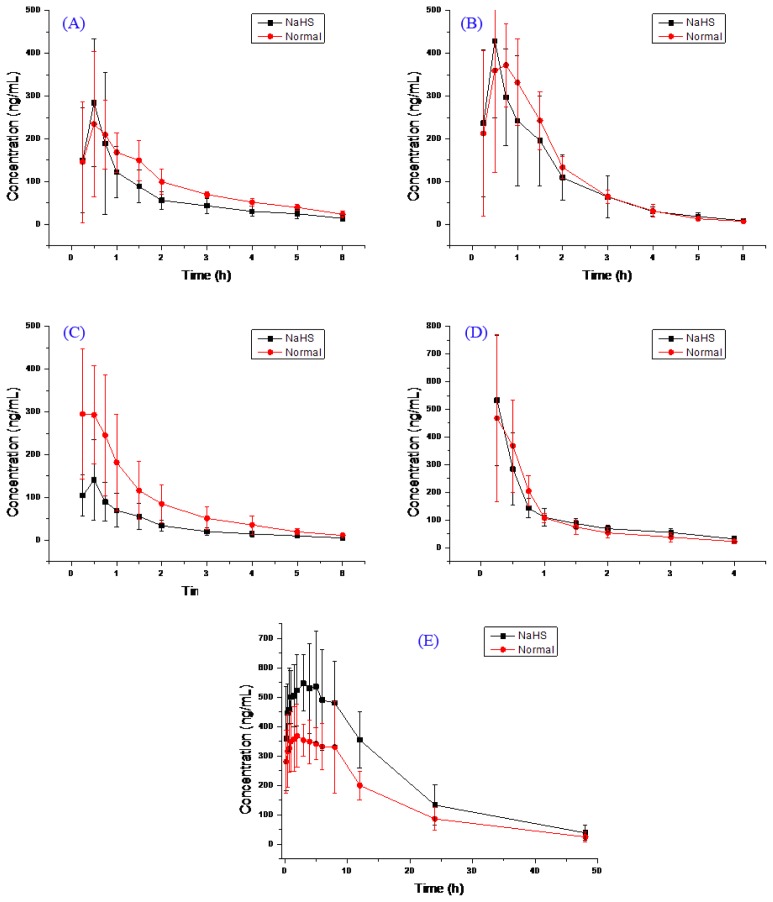
The pharmacokinetics profiles of bupropion (**A**); metroprolol (**B**); midazolam (**C**); omeprazole (**D**); and tolbutamide (**E**).

The main pharmacokinetic parameters after oral administration of bupropion, metroprolol, midazolam, omeprazole and tolbutamide from non-compartment model analysis are summarized in Table 1. The representative bupropion, metroprolol, midazolam, omeprazole and tolbutamide concentration *vs*. time profiles of twelve rats are presented in Figure 1.

As could be seen from Table 1, compared H_2_S group with the control group, the pharmacokinetic parameters of bupropion had changed, *t*_1/2_ from the 1.9 increased to 2.3 h, but there no statistical significance (*p* > 0.05), *AUC*_(0–_*_t_*_)_ from the 532.7 reduced to 393.0 ng/mL h with no significant difference (*p* > 0.05); *CL* from 13.7 reduced to 21.7 L/h/kg, but there was no significant difference (*p* > 0.05); *C*_max_ varied from 279.2 to 308.4 ng/mL, but there was no significant difference (*p* > 0.05). Compared to the control group, the H_2_S group, *AUC*_(0–_*_t_*_)_ reduced, *CL* increased, however, there was no significant difference for these pharmacokinetic parameters (*p* > 0.05), which indicates that the H_2_S will not induce or inhibit the activity of CYP2B6 enzyme.

Similar results were found in metoprolol, the pharmacokinetic parameters of metoprolol had changed between control group and H_2_S group, but there was no significant difference (*p* > 0.05), which shows that the H_2_S would not induce or inhibit the activity of CYP2D6 enzyme.

Compared H_2_S group with the control group, the pharmacokinetic parameters of midazolam had changed, *AUC*_(0–_*_t_*_)_ from the 509.2 reduced to 210.0 ng/mL h, there was significant difference (*p* < 0.05); *CL* from 18.6 increased to 44.6 L/h/kg, there was significant difference (*p* < 0.05); *C*_max_ varied from 330.9 to 152.9 ng/mL, there was no significant difference (*p* > 0.05). Compared to the control group, the H_2_S group, *AUC*_(0–_*_t_*_)_ reduced (*p* < 0.05), *CL* increased (*p* < 0.05), *C*_max_ becomes lower, which indicates that the H_2_S could induce the activity of CYP3A4 enzyme.

The pharmacokinetic parameters of omeprazole had almost not changed between control group and H_2_S group, and there was no significant difference for these pharmacokinetic parameters (*p* > 0.05), which indicates that the H_2_S would not induce or inhibit the activity of CYP2C19 enzyme.

Compared H_2_S group with the control group, the pharmacokinetic parameters of tolbutamide had changed, *CL* from the 0.11 h reduced to 0.08 h, and there was statistical significance (*p* < 0.05), *AUC*_(0–_*_t_*_)_ from the 6799.3 increased to 10,429.3 ng/mL h with significant difference (*p* < 0.05); *C*_max_ varied from 484.7 to 640.4 ng/mL, and there no statistical significance (*p* > 0.05). Compared to the control group, H_2_S group, *AUC*_(0–_*_t_*_)_ increased (*p* < 0.05), *CL* reduced (*p* < 0.05), *C*_max_ became higher, showing that the H_2_S would inhibit the activity of CYP2C9 enzyme.

As could be seen from Figure 1, the *AUC* of bupropion and omeprazole H_2_S group is the almost same as the control group, the *AUC* and *C*_max_ tolbutamide in H_2_S group is higher than the control group, the *AUC* and *C*_max_ of midazolam in H_2_S group is lower than the control group, this result is consistent with Table 1.

NaHS can be decomposed into Na^+^ and HS^−^*in vivo*, it will form H_2_S when HS^−^ combine with H^+^*in vivo*. 5 mg/kg NaHS given to the rats, the level of H_2_S in exposed individuals may be about 89.2 μmol/kg H_2_S. H_2_S could not influence the activities of CYP450 isoforms CYP2B6, CYP2D6, CYP2C19 *in vivo* in rats, while H_2_S could induce the activity of CYP3A4 and inhibit the activity of CYP2C9 *in vivo* in rats. It has certain guiding significance to the clinical treatment, when the drugs metabolism through CYP2C9 orally combined with NaHS, we must pay attention to dosage, appropriate reduce the dose, the plasma drug concentration may be too high and poisoning. When the drugs metabolism through CYP3A4 orally combined with NaHS, should increase the dose, so as not to hinder the effect of drugs. However, the human case is not the same as rat, the result is only for clinical reference.

## 3. Material and Methods

### 3.1. Chemicals and Reagents

Bupropion, metroprolol, midazolam, omeprazole and tolbutamide (all >98%) and the internal standard carbamazepine (IS) were purchased from Sigma-Aldrich Company (St. Louis, MO, USA). HPLC grade acetonitrile and methanol were purchased from Merck Company (Darmstadt, Germany). Ultra-pure water (resistance >18 mΩ) prepared by a Millipore Milli-Q purification system (Bedford, MA, USA).

### 3.2. Animals

Male Sprague-Dawley rats (250 ± 20 g) were obtained from Shanghai SLAC Laboratory Animal Co., Ltd. (Shanghai, China). The animal license number was SCXK (Shanghai, China) 2012–0005. All twenty rats were housed at Wenzhou Medical University Laboratory Animal Research Center (Wenzhou, China). Animals were housed under controlled conditions (22 °C) with a natural light-dark cycle. All experimental procedures were conducted according to the Institutional Animal Care guidelines (Laboratory Animal Center of Wenzhou Medical University, Wenzhou, China) and approved ethically by the Administration Committee of Experimental Animals, Laboratory Animal Center of Wenzhou Medical University.

### 3.3. Instrumentation and Conditions

All analysis was performed with a 1200 Series liquid chromatograph (Agilent Technologies, Waldbronn, Germany) equipped with a quaternary pump, a degasser, an autosampler, a thermostatted column compartment, and a Bruker Esquire HCT mass spectrometer (Bruker Technologies, Bremen, Germany) equipped with an electrospray ion source and controlled by ChemStation software (Version B.01.03 (204), Agilent Technologies, Waldbronn, Germany).

Chromatographic separation was achieved on a 150 mm × 2.1 mm, 5 μm particle, Agilent Zorbax SB-C18 column at 30 °C. A gradient elution programme was conducted for chromatographic separation with mobile phase A (0.1% formic acid in water) and mobile phase B (acetonitrile) as follows: 0–4.0 min (10%–80% B), 4.0–8.0 min (80%–80% B), 8.0–9.0 min (80%–10% B), 9.0–13.0 min (10%–10% B). The flow rate was 0.4 mL/min.

The quantification was performed by the peak-area method. The determination of target ions were performed in selective ion monitoring mode (*m*/*z* 240 for bupropion, *m*/*z* 268 for metroprolol, *m*/*z* 326 for midazolam, *m*/*z* 198 for omeprazole, and *m*/*z* 237 for IS) in positive ion electrospray ionization interface. Drying gas flow was set to 7 L/min and temperature to 350 °C. Nebuliser pressure and capillary voltage of the system were adjusted to 25 psi and 3500 V.

### 3.4. Preparation of Standard Solutions

Stock solutions of 1.0 mg/mL each of bupropion, metroprolol, midazolam, omeprazole, tolbutamide and IS were prepared in methanol. The working standard solutions of each analyte were prepared by serial dilution of the stock solution with methanol. All of the solutions were stored at 4 °C and brought to room temperature before use.

The calibration standards were prepared by spiking blank rat plasma with appropriate amounts of bupropion, metroprolol, midazolam, omeprazole and tolbutamide. Calibration plots of each probe drug were constructed in the range 10–2000 ng/mL for plasma (10, 20, 50, 100, 200, 500, 1000 and 2000 ng/mL). Quality-control (QC) samples were prepared by the same way as the calibration standards, three different plasma concentrations (20, 400, and 1600 ng/mL). The analytical standards and QC samples were stored at −20 °C.

### 3.5. Pharmacokinetic Study

Twelve male Sprague-Dawley rats (250 ± 20 g) were randomly divided to control group and H_2_S group (*n* = 6). Control group were give saline by oral administration once a day for 7 days; while H_2_S group were give NaHS (5 mg/kg) by oral administration once a day for 7 days. After complete the modeling, the H_2_S and control group were given the mixtured five probe drugs by oral administration, the dose of bupropion, metroprolol, midazolam, omeprazole and tolbutamide were 8, 8, 8, 8 and 0.8 mg/kg, respectively.

Blood samples (0.3 mL) were collected from the tail vein into heparinized 1.5 mL polythene tubes at 5, 15, 30 min and 1, 1.5, 2, 3, 4, 6, 8, 12, 24, 36, 48 h after oral administration of five probe drugs. The samples were immediately centrifuged at 8000 r/min for 5 min, and 100 μL plasma was obtained for each sample.

The plasma samples were extracted and measured by LC-MS. In a 1.5 mL centrifuge tube, an aliquot of 10 μL of the internal standard working solution (2.0 μg/mL) was added to 0.1 mL of collected plasma sample followed by the addition of 0.2 mL of acetonitrile. After the tube was vortex-mixed for 1.0 min, the sample was centrifuged at 15,000 r/min for 10 min. The supernatant (2 μL) was injected into the LC-MS system for analysis.

Plasma probe drugs concentration versus time data for each rat was analyzed by DAS software (Version 3.0, Drug Clinical Research Center of Shanghai University of Traditional Chinese Medicine and Shanghai BioGuider Medicinal Technology Co., Ltd., Shanghai, China). The pharmacokinetic parameters of probe drugs of the H_2_S group and control group with the *t*-test inspection were analyzed by SPSS 16.0 statistical software (SPSS Inc., Chicago, IL, USA). A *p* < 0.05 was considered as statistically significant.

## 4. Conclusion

H_2_S could not influence the activities of CYP450 isoforms CYP2B6, CYP2D6, CYP2C19 *in vivo* in rats, while H_2_S could induce the activity of CYP3A4 and inhibit the activity of CYP2C9 *in vivo* in rats. This has certain guiding significance to clinical treatment. In the case of about 16% of common drugs’ metabolism through CYP2C9, when these drugs are orally combined with NaHS, we must pay close attention to dosage and appropriately reduce the dose of the drug, otherwise, the plasma drug concentration may be too high and have a poisoning effect. Furthermore, in the case of about 50% of prescription drugs metabolism through CYP2C9, when orally combined with NaHS, the dose must be increased, so as not to hinder the effect of the drug. However, it is noted that the human case differs from rat, therefore, the result is only provided for clinical reference.

## Figures and Tables

**Table 1.** Pharmacokinetic parameters of bupropion, metroprolol, midazolam, omeprazole and tolbutamide (mean ± SD), Control-group *n* = 6, H_2_S-group *n* = 6.

Compound	Group	*AUC* (0–*t*) ng/mL h	*AUC* (0–*∞*) ng/mL h	MRT (0–*t*) h	MRT (0–*∞*) h	*t*_1/2_ h	*T*_max_ h	*CL* L/h/kg	*V* L/kg	*C*_max_ ng/mL
Bupropion	H_2_S	393.0 ± 163.5	451.1 ± 176.3	1.8 ± 0.3	2.8 ± 1.3	2.3 ± 1.0	0.5 ± 0.1	21.7 ± 12.7	68.9 ± 40.3	308.4 ± 164.9
	Normal	532.7 ± 141.4	603.6 ± 123.0	2.1 ± 0.3	3.0 ± 1.0	1.9 ± 0.7	0.8 ± 0.4	13.7 ± 2.8	39.3 ± 17.9	279.2 ± 119.3

Metoprolol	H_2_S	627.7 ± 262.9	641.3 ± 265.9	1.5 ± 0.1	1.7 ± 0.1	1.1 ± 0.2	0.5 ± 0.1	14.3 ± 5.8	24.2 ± 12.1	447.1 ± 188.5
	Normal	693.8 ± 172.4	704.6 ± 168.7	1.5 ± 0.3	1.6 ± 0.3	1.0 ± 0.3	0.7 ± 0.2	12.0 ± 3.0	18.2 ± 8.2	463.3 ± 139.1

Midazolam	H_2_S	210.0 ± 95.5 *	225.0 ± 100.7 *	1.7 ± 0.3	2.2 ± 0.6	1.7 ± 0.3	0.4 ± 0.1	44.6 ± 25.8 *	114.6 ± 92.6	152.9 ± 91.5
	Normal	509.2 ± 244.8	535.1 ± 239.4	1.6 ± 0.1	2.2 ± 0.7	1.7 ± 1.1	0.3 ± 0.1	18.6 ± 10.5	58.1 ± 73.1	330.9 ± 117.1

Omeprazole	H_2_S	446.7 ± 90.2	540.9 ± 90.5	1.2 ± 0.3	2.2 ± 0.7	1.9 ± 0.4	0.3 ± 0.2	15.2 ± 2.7	41.3 ± 11.8	536.7 ± 226.7
	Normal	427.4 ± 155.2	479.9 ± 165.4	1.1 ± 0.2	1.7 ± 0.4	1.7 ± 0.6	0.4 ± 0.2	18.1 ± 5.0	43.9 ± 21.5	491.0 ± 276.0

Tolbutamide	H_2_S	10,429.3 ± 2871.6 *	11,099.1 ± 3218.4 *	12.2 ± 2.7	14.8 ± 4.0	10.1 ± 2.7	2.9 ± 1.4	0.08 ± 0.02 *	1.1 ± 0.3	640.4 ± 162.4
	Normal	6799.3 ± 769.0	7261.5 ± 916.4	12.7 ± 1.9	16.0 ± 5.8	10.6 ± 4.7	3.6 ± 3.2	0.11 ± 0.02	1.6 ± 0.6	484.7 ± 98.3

Compared with the control group,

* : *p* < 0.05.
